# Orchestrating Self‐Replication in Artificial Cells with Digital Microfluidics

**DOI:** 10.1002/smll.202509316

**Published:** 2025-11-18

**Authors:** Guanzhong Zhai, Pantelitsa Dimitriou, Jason Sengel, Mark Ian Wallace

**Affiliations:** ^1^ Department of Chemistry King's College London London SE1 1DB United Kingdom; ^2^ The Francis Crick Institute 1 Midland Road London NW1 1AT United Kingdom

**Keywords:** artificial cells, digital microfluidics, self‐replication

## Abstract

A defining feature of living cells is their ability to self‐replicate; but creating artificial cells with this capability remains challenging, due to the complexity of biological division machinery. Rather than seeking to reconstitute this machinery, direct control of DNA replication and compartment division using digital microfluidics (DMF). This approach allows us to precisely orchestrate these two fundamental processes, providing insight into how they must be coupled for successful self‐replication. The system achieves controlled cycles of replication and division, with daughter compartments inheriting parental DNA and maintaining genetic continuity across multiple generations ‐ a key feature of living systems that has been difficult to achieve in artificial cells. By implementing these processes through direct physical manipulation rather than biochemical complexity, a simple testbed is provided that will help to disentangle the essential requirements for self‐replicating systems.

## Graphical abstract

This work demonstrates controlled DNA replication and compartment division using digital microfluidics, enabling artificial cells to undergo repeated self‐replication cycles. The system couples physical manipulation with genetic continuity across generations, offering a simplified platform to explore the essential principles of self‐replicating living systems.

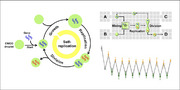

## 1 Introduction

Artificial cells represent a major bottom‐up approach to synthetic biology, with the goal of understanding living systems and mimicking their characteristics.^[^ ^1^, ^2^, ^3^ ^]^ Recent advances have demonstrated artificial cells that exhibit key life‐like characteristics, including genetic information transfer,^[^ ^4^ ^]^ metabolism,^[^ ^5^ ^]^ compartmentalisation,^[^ ^3^ ^]^ cell growth and division,^[^ ^6^ ^]^ and communication and motility.^[^ ^7^ ^]^ Despite recent advancements, integrating all of these complex functions and establishing a continuous cell cycle of artificial cell replication remains a significant challenge.^[^ ^8^ ^]^ Current approaches to compartmentalise DNA replication generally rely on biochemical mechanisms including protein‐mediated membrane remodeling,^[^ ^9^ ^]^ phase separation,^[^ ^10^ ^]^ or chemical reactions that generate membrane components.^[^ ^11^ ^]^ While these approaches have provided remarkable insights, they are frequently associated with low efficiency, poor control, and difficulty in achieving reliable coordination with DNA replication. To make progress, we reasoned that a system that enables explicit control over both replication and division would allow us to better understand the essential coupling between these processes, a fundamental question in understanding the minimal requirements for life‐like systems. Thus here we sought to establish a DMF approach to directly control compartmentalisation and produce simple cell cycles capable of propagating genetic information.

Vesicle‐based artificial cells have achieved DNA self‐replication by encapsulating the appropriate constituents and exposing them to the required amplification temperatures.^[^ ^12^, ^13^, ^14^ ^]^ Integrating gene replication with membrane dynamics can also enable the propagation of genetic information and the formation of spontaneous compartments.^[^ ^11^ ^]^ However, vesicle division often requires extrinsic triggers, such as compositional changes of vesicle precursors,^[^ ^15^ ^]^ environmental modifications (e.g., illumination in the presence of photosensitive components^[^ ^16^ ^]^), osmotic pressure changes^[^ ^17^ ^]^ to enhance vesicle fission, or pH shifts to drive vesicle growth.^[^ ^18^, ^19^ ^]^ Notably, Kurihara et al. demonstrated self‐assembly and DNA replication in giant unilamellar vesicles, where growth and division were driven by lipid precursor self‐assembly;^[^ ^20^ ^]^ although DNA replication over several cycles was reported, limitations were a lack of timing and size control during division. Abil et al. also recently encapsulated a DNA self‐replicator in liposomes, where the DNA template could sustain self‐encoded replication over >10 rounds and carry out adaptive evolution through a recombinant gene expression system;^[^ ^21^ ^]^ freeze‐thaw cycles were used to achieve vesicle fusion and fission that requires redistribution of DNA across the vesicle population between evolutionary rounds.

Artificial cells based on coacervates have also shown spontaneous division and growth,^[^ ^22^ ^]^ but predominantly without controlled inheritance of genetic information. For example, Dreher et al. demonstrated DNA‐containing coacervates that could undergo division while maintaining DNA content but without control over the timing and coordination of these processes.^[^ ^23^ ^]^ Coacervate droplets formed by in situ oligonucleotide ligation and elongation^[^ ^24^ ^]^ suitable for Darwinian evolution^[^ ^25^ ^]^ have also been reported, and more recently protocell growth induced by a compartmentalised DNA replication reaction.^[^ ^26^ ^]^

DNA amplification within water‐in‐oil droplets have also been reported.^[^ ^27^, ^28^ ^]^ For example, Sakatani et al. developed a simplified DNA replication system combining rolling‐circle isothermal replication of DNA encoding a DNA polymerase (and externally supplied recombinase), in the presence of in vitro transcription and translation reagents to generate the encoded polymerase.^[^ ^29^ ^]^ Thus although DNA self‐replication in artificial cells is arguably well established, coordinating compartment division remains a challenge. For example, liposomes require some form of transmembrane channel to realise transport^[^ ^30^ ^]^ and are often unable to maintain the encapsulation for prolonged periods.^[^ ^31^, ^32^ ^]^ Coacervate's advantage in lacking a phospholipid membrane also leads to restrictions in division due to surface tension requirements.^[^ ^33^ ^]^ Similarly, emulsion systems are also membraneless, but have limited stability and size control in the absence of appropriate surfactant concentrations^[^ ^34^ ^]^ and lack control of division and growth.^[^ ^27^ ^]^

Overall, each of these methods possesses contrasting challenges that have hampered progress toward the reliable replication of genetic material and cell volume require to build a bottom‐up minimal self‐replicating cell. We reasoned that DMF provides an alternative route to directly control the size and shape of individual aqueous droplets and might serve as an alternative basis for artificial cell compartments.^[^ ^35^, ^36^ ^]^ In DMF, the electrowetting‐on‐dielectric (EWOD) effect changes surface wettability, resulting in controlled actuation of droplet position.^[^ ^37^, ^38^ ^]^ DMF has low sample volume, digital control and flexible programmability, making it suitable for a wide range of biological applications ‐ including single‐cell proteomic,^[^ ^39^ ^]^ cell invasion,^[^ ^40^ ^]^ and cell culture.^[^ ^41^ ^]^ Notably, DMF has revolutionised nucleic acid research, finding applications in DNA storage,^[^ ^42^ ^]^ RNA extraction,^[^ ^43^ ^]^ and DNA amplification.^[^ ^44^ ^]^ Relevant to this work, Ruan et al. demonstrated amplification of DNA isolated from single cells within DMF droplets,^[^ ^45^ ^]^ and Kalsi et al. used DMF‐based DNA amplification for gene detection.^[^ ^46^ ^]^

**Figure 1:**
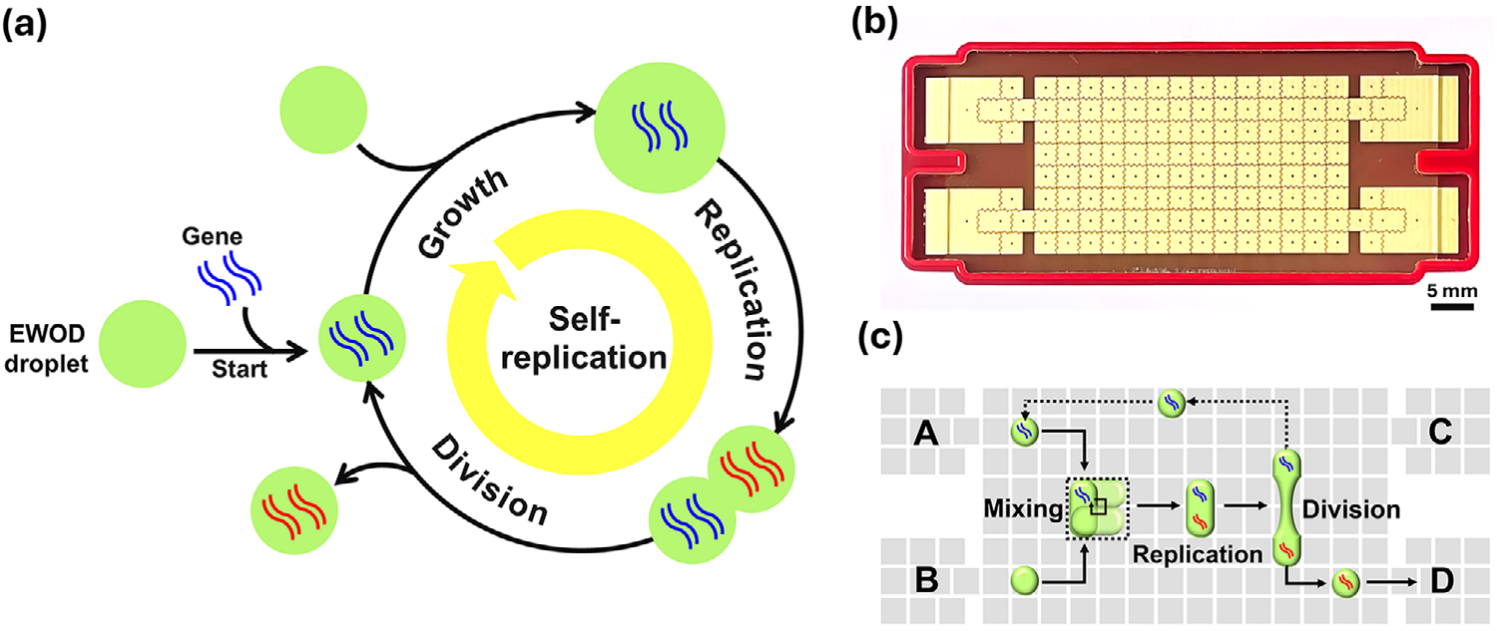
DMF‐based DNA self‐replication. a) Schematic representation of the steps for DNA self‐replication within EWOD droplets: DNA encapsulation, droplet growth, DNA replication within the droplet, and droplet division. b) Photograph of the OpenDrop chip. Scale bar: 5 mm. OpenDrop comprises a rectangular array of 112 gold‐coated electrodes, plus 4 input/output EWOD reservoirs. c) Schematic diagram illustrating the replication process: Droplets containing reagents are combined and mixed to generate the droplet growth step. DNA replication is then achieved under temperature controlled conditions using resistive heater build into the array. The droplet is then divided equally. One daughter droplet is collected, while the other returns to the starting point to initiate the next round of self‐replication.

Here, we adapt the open‐source DMF platform OpenDrop^[^ ^47^ ^]^ to generate cycles of DNA replication and compartment division (**Figure** **1**b). OpenDrop has been used to study areas as varied as the self‐assembly of multifunctional magnetic nanoclusters,^[^ ^48^ ^]^ in situ spectroscopy,^[^ ^49^ ^]^ and cell‐free protein expression and plasmid screening.^[^ ^50^ ^]^

Figure 1a illustrates our overall strategy, in which genetic material is encapsulated inside an EWOD droplet and replication is initiated via programmed fusion with a second droplet containing the necessary components for DNA replication. The sequence of mixing, DNA replication, droplet division, and fusion then creates a cycle of continuous self‐replication (Figure 1c). Through engineering of surface properties and temperature control, we have optimized the reliable DNA amplification within droplet‐based cell mimics that can be precisely divided using EWOD actuation. Daughter droplets inherit the parental DNA and maintain replication capacity, enabling sustained multiple generations of self‐replication. This system demonstrates a path toward artificial cells that can grow and multiply through direct physical control rather than biochemical complexity, providing insights into the minimal physical principles required for sustained self‐replication.

## 2 Results and Discussion

### 2.1 DMF Droplet Replicator Design

**Figure 2:**
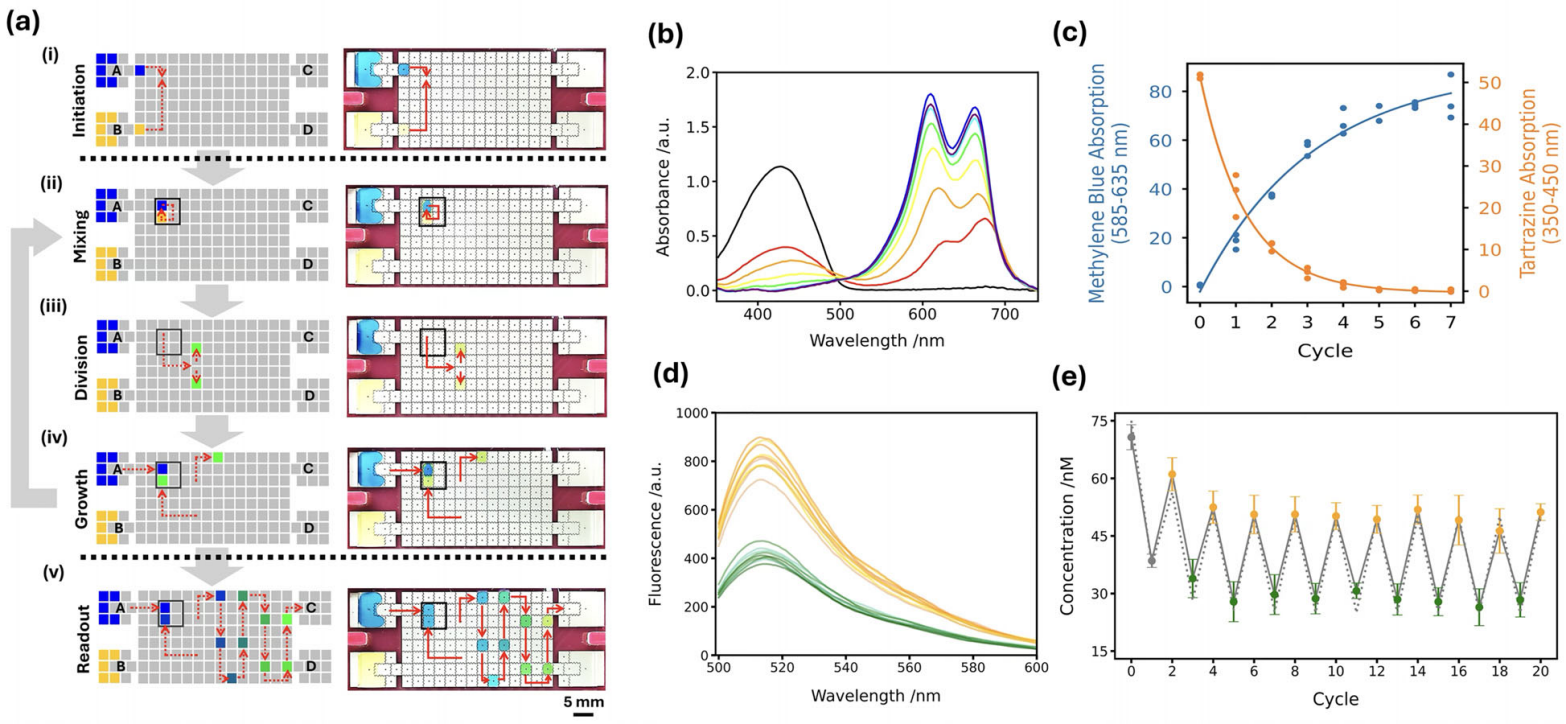
DMF cyclic replication. a) Schematic (left) of the predefined electrode paths designed to generate droplet replication cycles. Red dashed lines indicate the paths followed by the droplets. Corresponding live camera images (right) are shown, where reservoirs A and B are filled with methylene blue and tartrazine, respectively. Scale bar: 5 mm. Droplet replication has five stages: i) Initiation: Reservoirs A and B each input a 2 µL droplet. ii) Mixing: Two droplets from the A and B reservoirs merged and repeatedly mixed for 20 rounds clockwise in a 2 × 2 electrode array. iii) Division: Droplets are divided into two via an electrode pattern designed to induce efficient division. iv) Growth: One daughter droplet is returned to the Mixing stage and combined with a fresh droplet from reservoir A. The second daughter droplet is collected in a queue and stored for collection. Queue spacing is designed to avoid merging. v) Readout: Droplets are sequentially output to reservoir C and collected using a gel loading pipette. b) Rounds of cyclic droplet propagation by mixing methylene blue (reservoir A) with tartrazine (reservoir B). The resultant output droplets were analysed and the absorption spectra from serial dilution cycles are represented by a rainbow color gradient (from red (× 1) to purple (× 7)). Absorbance peaks at 427 and 664 nm correspond to tartrazine and methylene blue, respectively. The black line represents cycle 0, where the tartrazine droplet was not diluted. c) Plotted relation between absorbance of tartrazine (orange) and methylene blue (blue) during droplet serial dilution cycles. d) Spectra from two‐state droplet cyclic replication (cycles 2–20) alternating between fluorescein (75 nM in 0.1 M Tris‐HCl, pH 8.0, 1% (v/v) silicone oil AR5) and buffer. Even‐numbered cycles (fluorescein, orange) and odd‐numbered cycles (buffer, green) exhibit spectral overlap. e) Two‐state replication over 20 cycles. Measured (grey, solid line) and calculated (grey, dotted line) concentrations of fluorescein plotted per cycle. Colored markers show mean concentration over 3 repeats: cycles 0 and 1 (grey), even‐numbered cycles (orange), odd‐numbered cycles (green). The error bars represent the standard deviation, *n* = 3.

We first tackled compartment division by establishing a minimal cycle of droplet volume replication in the absence of DNA (**Figure** **2**). Replication cycling requires a programmed sequence of voltages to the electrodes in our array control the droplet position. Figure 2a illustrates our final programmed sequence plus corresponding live camera images. Our minimal replication cycle comprised five stages: i) **Initiation**: Reservoirs A and B each input a 2 µL droplet; ii) **Mixing**: Droplets from A and B reservoirs were merged and repeatedly mixed (20 rounds clockwise mixing of a 2 × 2 square array; iii) **Division**: Droplets were then divided using a division array pattern (Figure S5a, Supporting Information) to create two equal sized daughters; iv) **Growth**: One daughter droplet is returned to the mixing step for a subsequent round of fusion with a ‘fresh’ droplet from reservoir A. The second daughter droplet was collected and placed in a serpentine output queue where each droplet was spaced by three electrodes to avoid merging; v) **Readout**: Finally the queue is read out, with individual droplets collected from the output reservoir using a gel loading pipette for further analysis.

### 2.2 DMF Replicator Validation by Serial Mixing

To illustrate DMF replicator action rounds of cyclic droplet propagation were conducted by mixing methylene blue (1 mM, ultra‐pure water, 1% v/v silicone oil AR5, reservoir A) with tartrazine (1 mM, ultra‐pure water, 1% v/v silicone oil AR5, reservoir B) (Figure 2a; Movie S1, Supporting Information) Two 2 µL droplets were generated ‐ one from reservoir A (methylene blue) and one from reservoir B (tartrazine), and then mixed. For ‘cycle 0’, a droplet containing only trartrazine was generated. Output droplets from seven replication cycles were collected and absorption spectra determined (Figure 2b). As expected, the characteristic absorption peak of tartrazine at 427 nm decreased and methylene blue at 664 nm increased as cycles repeated. During the replication cycles, methylene blue showed an exponential increase in absorbance, whereas tartrazine decreased exponentially and reached a plateau from cycle 5 onward (Figure 2c). The distinct trends of methylene blue and tartrazine are consistent with the expected serial mixing during the droplet replication cycles.

To further assess platform reproducibility, we also repeated cycles of mixing between an aqueous droplet of fluorescein (75 nM, 0.1 M Tris‐HCl, pH 8.0, 1% (v/v) silicone oil AR5) and a droplet of buffer alone (0.1 M Tris‐HCl, pH 8.0, 1% (v/v) silicone oil AR5). Output droplet composition was then varied by alternating the introduced droplet between aqueous buffer and the fluorescein reservoirs. The output droplets were extracted and the fluorescence spectra were measured (Figure 2d). By calibrating measured fluorescence maxima against standardized fluorescein solutions (Figure S6b, Supporting Information) we can plot output droplet concentration over 20 cycles (Figure 2e). Measured (grey solid line) and calculated (grey dashed line) concentrations after each programmed cycle are depicted. Figure S7 (Supporting Information) shows the linear dependence between the measured and expected values. Across 5 independent repeats we measure a coefficient of variation of 3.72 % (27.39 ± 1.02 nM) (Figure S8b, Supporting Information) and 4.65 % (15.68 ± 0.73 nM) (Figure S8d, Supporting Information), demonstrating good reproducibility.

### 2.3 DMF DNA Replication

To develop a self‐sustaining cycle of DNA replication, we next established conditions for effective DMF replication of DNA using recombinase polymerase amplification (RPA). We selected RPA as it operates at mild temperatures (37–42 °C), requires minimal sample preparation, and delivers rapid amplification.^[^ ^51^, ^52^, ^53^ ^]^ In the RPA reaction mechanism, a recombinase ATPase (RecA) forms complexes with primers that facilitate their binding to complementary sequences on the target gene. After binding, RecA dissociates allowing DNA polymerase to initiate DNA synthesis; simultaneously, single‐stranded binding protein (SSB) stabilizes the exposed DNA strands to prevent re‐annealing.^[^ ^54^ ^]^ The low operating temperature of RPA minimizes sample evaporation, making it particularly suitable for the low sample volumes present in microfluidic applications.^[^ ^46^, ^55^ ^]^ We used a commercial fluorescent reporter (TwistAmp Exo, TwistDx) in which fluorescent signals are generated using a fluorophore/quencher ‘probe’ that is complementary to the target DNA.^[^ ^54^, ^56^ ^]^ The probe also contains a tetrahydrofuran site that is a substrate for Exonuclease III ‐ cutting the probe when complexed with target DNA separates the fluorophore/quencher complex and generates a fluorescent signal.^[^ ^46^ ^]^

**Figure 3:**
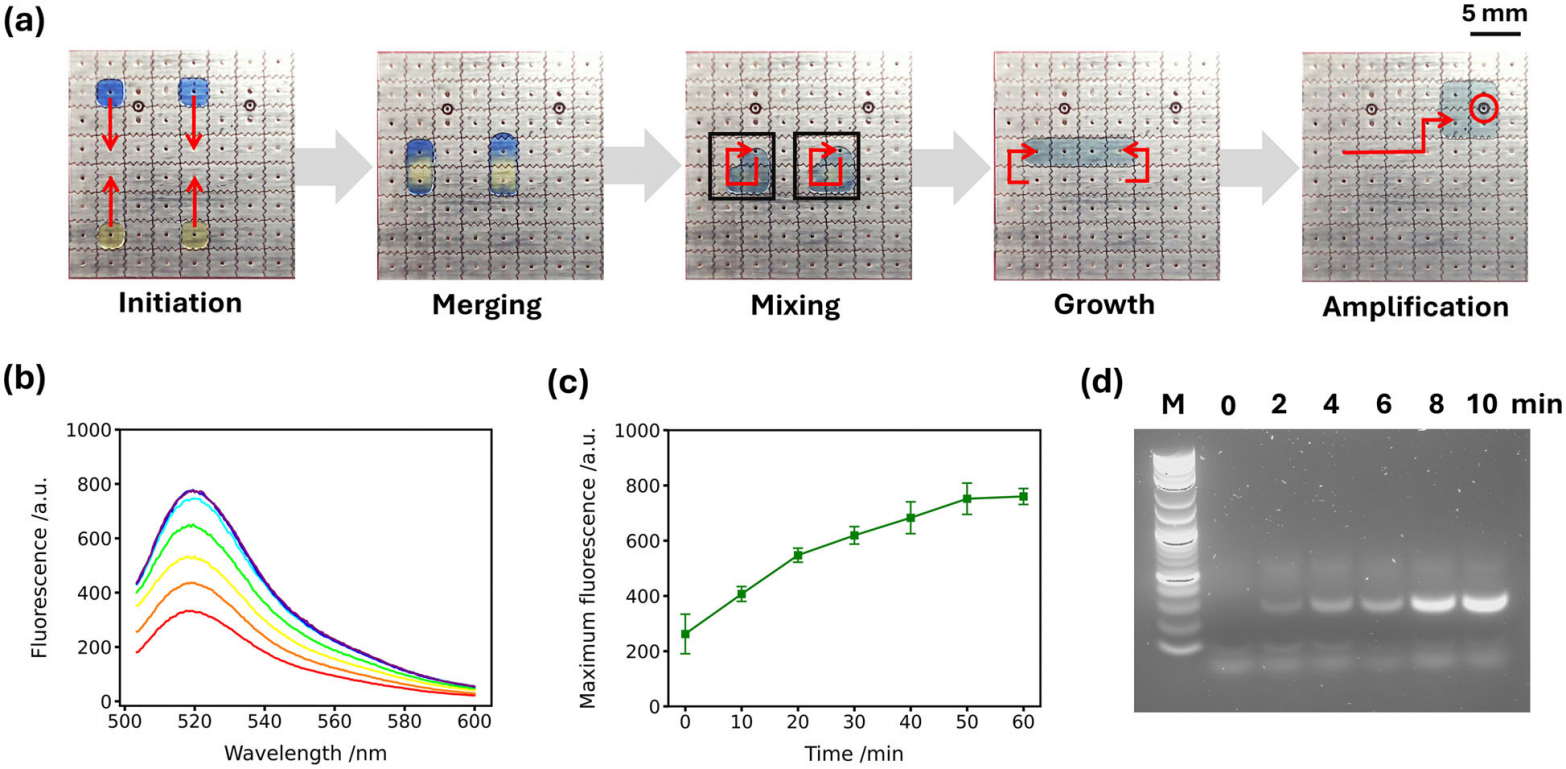
DMF RPA amplification. a) Schematic of the steps required for on‐chip RPA. Methylene blue and tartrazine (yellow) have been added to the droplets in these images to improve visibility. Initiation: Two (blue) droplets are created from the RPA mixture and two (yellow) droplets from the Mg(OAc)_2_ solution. Merging and Mixing: Blue and yellow droplets were merged, and mixed within two square regions. Growth: Four green droplets merged together after the completion of mixing. Amplification: Droplets were heated to 40 °C on the chip heater (red circle). b) Representative fluorescence spectra of RPA reaction on a chip. 0 min (red), 10 min (orange), 20 min (yellow), 30 min (green), 40 min (cyan), 50 min (blue), 60 min (purple). c) Kinetics of RPA reaction, reporting fluorescence maxima every 10 min. The error bars represent the standard deviation, *n* = 3. d) Agarose gel electrophoresis of RPA kinetic products from pUC19.

As RPA requires incubation at mild temperatures (37–42 °C) for 50 min (**Figure** **3**c), preventing aqueous droplet evaporation is critical to avoid reagent concentration bias. To address this, we optimized OpenDrop for rapid DMF actuation of aqueous droplets not in air (prone to evaporation at incubation temperatures), but in an oil bulk phase (octamethyltrisiloxane, 1 cSt) — where droplet volume stability is maintained by eliminating evaporation. Direct experimental evidence for this optimization is provided in Figure S9 (Section S2.2, Supporting Information). Silicone oil is commonly used in DMF systems to prevent evaporation.^[^ ^45^, ^57^ ^]^ For example, Shen et al. surrounded DNA amplification droplets with 2 cSt silicone oil, but we found that with a viscosity of 1 cSt, octamethyltrisiloxane has a kinematic viscosity closer to that of water of water (0.89 cSt,^[^ ^58^ ^]^), providing superior droplet mobility and division into daughter droplets. To achieve efficient droplet actuation, we also made a number of additional improvements: engineering a spacer to maintain a consistent distance between the electrode array and the ITO cover glass, and optimizing the thickness and composition of the polymer film coatings (Section S2.2, Figures S1– S3, Supporting Information).

Droplets were dispensed from reservoir A (RPA reaction components: 0.84 µM forward primer, 0.84 µM reverse primer, 0.24 µM probe, 0.32 nM positive control DNA template; 50 mM Tris‐HCl, pH 7.9; 200 µM dNTPs; 3 mM ATP; 100 mM potassium acetate; 2 mM DTT; 50 mM phosphocreatine; 0.1% Tween 20 (v/v); a lyophilized enzyme pellet containing recombinase, SSB, strand‐displacing polymerase, and ExoIII) and reservoir B (28 mM magnesium acetate; 50 mM Tris‐HCl, pH 7.9; 200 µM dNTPs; 3 mM ATP; 100 mM potassium acetate; 2 mM DTT; 50 mM phosphocreatine). Droplets were merged and mixed (Figure 3a), initiating RPA. The mixed droplet was moved to a resistive heating element and maintained at 40 °C (Figure 3a). RPA was monitored by collecting the output droplets at 10‐min intervals and measuring the fluorescence response of the probe (Figure 3b). To monitor amplification kinetics, a series of independent DMF RPA reactions were performed for time points: 0, 10, 20, 30, 40, 50, and 60 min. For each time point, fresh reaction droplets were dispensed from reservoirs A and B, respectively, merged and mixed to initiate amplification, and then incubated at 40 °C. The amplified products were collected and analyzed. Figure 3c shows that the reaction increases, reaching a plateau (50 min) as reagents are consumed. We also repeated the DMF reaction without exonuclease to allow gel electrophoresis of the DNA products.^[^ ^59^, ^60^ ^]^ Agarose gel electrophoresis of on‐chip RPA products confirms a time‐dependent amplification of DNA (Figure 3d). Here, a 277 bp segment of pUC19, was amplified as previously described,^[^ ^61^ ^]^ and within 10 min the amplification was significantly magnified (brightest bands), while sustaining the length of the DNA.

### 2.4 Sustained DMF DNA Replication in an Artificial Cell Cycle

**Figure 4:**
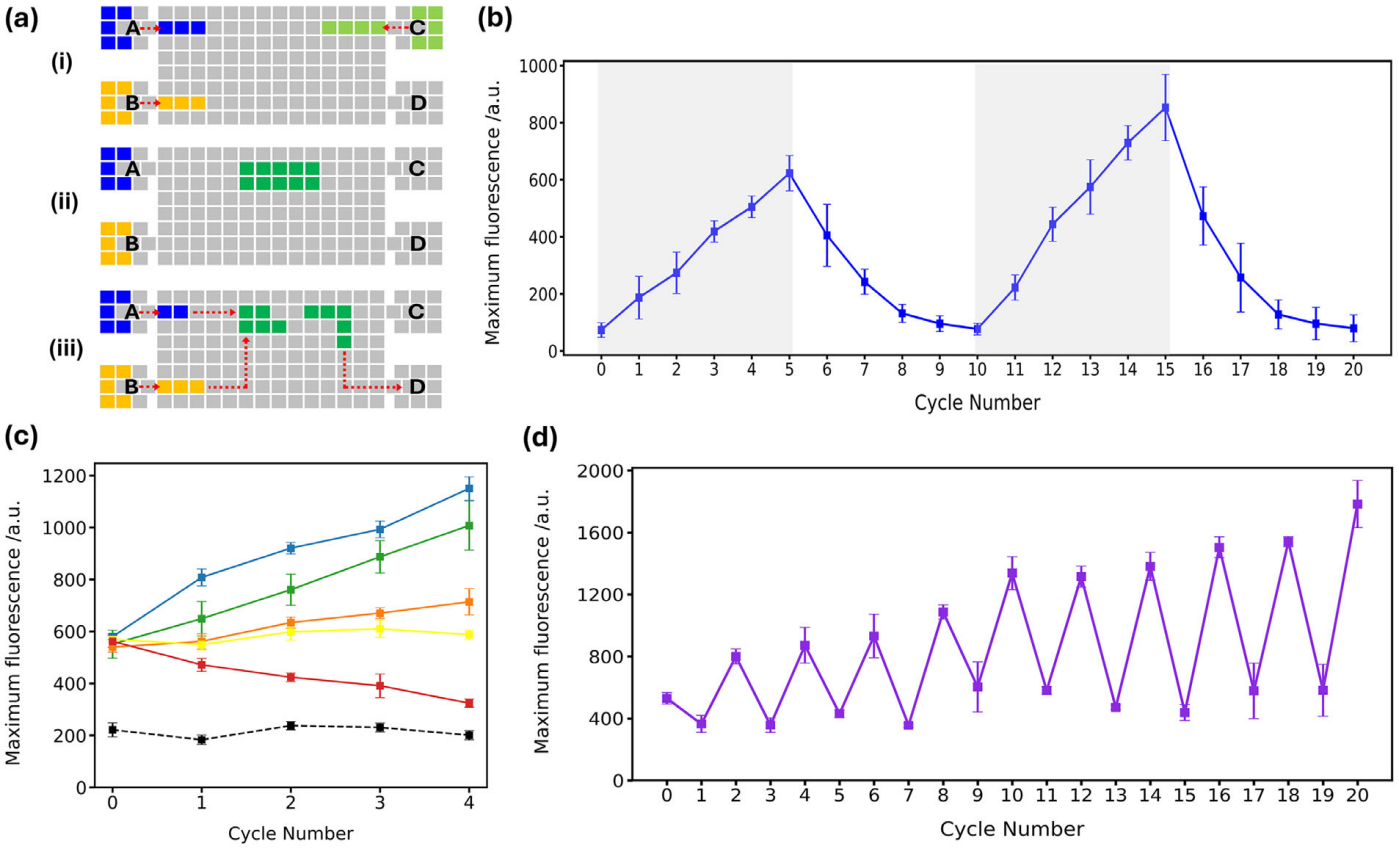
Regulation of the DNA self‐replication. a) Schematic of DMF program for DNA self‐replication: i) 6 µL droplets were dispensed from Reservoirs A (RPA reaction mixture) and B (Mg(OAc)_2_ solution), respectively. Reservoir C input 8 µL droplet of the DNA template only in cycle 0. ii) The 20 µL mixture was mixed and transported to a heating element for incubation (no heating in cycle 0). iii) The reaction droplet was divided into two equal 10 µL daughter droplets. One was collected via Reservoir D, and the other entered the next cycle. The remaining daughter droplet was mixed with fresh inputs: 4 µL from Reservoir A and 6 µL from Reservoir B, mixed and heated at 40 °C. b) Periodic addition of Mg(OAc)_2_ (Reservoir B, cycles 1–5 and 11–15) and EDTA (Reservoir B, cycles 6–10 and 16–20) enabled regulated amplification and attenuation of DNA. Fluorescence increased during Mg(OAc)_2_ addition (grey area) and decreased during EDTA addition due to inhibited amplification. The error bars represent the standard deviation, *n* = 3. (c) Fluorescence intensity corresponding DMF self‐replication cycles at different temperatures. 25 °C (red), 30 °C (yellow), 35 °C (orange), 45 °C (green), and 55 °C (blue). A baseline control, consisting of the reaction mixture without a DNA template, was maintained at the optimal temperature of 40 °C (black dashed line). The error bars represent the standard deviation, *n* = 3. d) Maximum fluorescence intensity during cycles of temperature switching. Cycle 0 represents the starting point without any amplification. For odd‐numbered cycles (cycle 1, cycle 3,… cycle 19), reaction temperature was set to 25 °C. For even‐numbered cycles (cycle 2, cycle 4,… cycle 20), the temperature was set to 45 °C. Each cycle supplies the same components and concentrations as in (a), stage (iii). The error bars represent the standard deviation, *n* = 3.

Finally, we combined these approaches to determine the conditions for sustained cycles of replication and division using DMF. We first quantified how Mg^2 +^ and EDTA activate and deactivate DMF RPA (Section S2.3, Supporting Information). **Figure** **4**a illustrates the DMF program. To initiate the DMF program, in cycle 0, an 8 µL droplet from the DNA solution (Reservoir C) was mixed with a 6 µL droplet of RPA reaction mixture and a 6 µL droplet of Mg(OAc)_2_ solution (Figure 4a(i)). The volume was then divided into two equal daughters, one of which was collected for analysis, the second entered cycle 1. In cycle 1, the cycle 0 daughter droplet was again combined with 4 µL from the RPA reaction mixture (Reservoir A), 6 µL of the Mg(OAc)_2_ solution (Reservoir B). The combined reaction volume was mixed, before moving to a resistive heating element and heated on chip at 40 °C for 15 min (Figure 4a(ii)). A total of 20 cycles were repeated, where the concentrations and volume of the RPA reaction mixture added in each cycle remained constant (to ensure the same supply of primers, probe, enzymes, ATP, and other components). A 6 µL of Mg(OAc)_2_ solution (56 mM magnesium acetate, 67.76 mM Tris‐HCl, 271.04 µM dNTPs, 4.07 mM ATP, 135.52 mM potassium acetate, 2.71 mM DTT, 67.76 mM phosphocreatine, pH 7.9) was added in cycles 1–5 and 11–15, and a 6 µL of EDTA solution (160 mM EDTA, 100 mM Tris‐HCl, pH 7.9) was supplied in cycles 6–10 and 16–20.

As shown in Figure 4b, fluorescence signals exhibit an increasing trend during cycles 1–5 and 11–15, indicating DNA amplification. During cycles 6‐10 and 16‐20, fluorescence signals decreased significantly, showing that EDTA effectively chelated Mg^2 +^, thus inhibiting DNA amplification, and reducing signals as DNA is serially diluted in each cycle. Each cycle demonstrates an integrated minimal cell cycle^[^ ^8^ ^]^ achieved through DMF actuation.

With a minimal DMF cell cycle established, we sought to optimize conditions to enable continuous DNA propagation over multiple cycles. As RPA is highly temperature dependent,^[^ ^54^ ^]^ we examined DNA self‐replication cycles at different temperatures as a means to control reaction propagation. To achieve this, we used the DMF program described in Figure 4a. Figure 4c shows the temperature dependence of fluorescence intensity, for the reagent concentrations and droplet volumes optimized in this example, at ≈40 °C the fluorescence intensity minimally increased, indicating a balance between RPA and the volume reduction upon cell division ‐ leaving the concentration of DNA in each cycle unchanged. At 25 °C, the activity of RPA enzymes is significantly inhibited (due to reduced reaction kinetics at low temperatures), resulting in no effective DNA amplification—thus, the serial dilution effect dominates, and fluorescence intensity decreases steadily with increasing cycle number. As temperature rises (30–45 °C), enzyme activity recovers: RPA amplification gradually outcompetes dilution. Based on these results, we then performed temperature‐switching experiments over 20 rounds: The experimental setup was identical to Figure 4a i)–iii). Temperature‐switching began from cycle 1, where the reaction temperature was set at 25 °C for odd‐numbered cycles (cycle 1, cycle 3, etc.), while for even‐numbered cycles (cycle 2, cycle 4, etc.), the temperature was maintained at 45 °C. Figure 4d shows continuous modulation of replication efficiency over the 20 cycles of DMF droplet division and replication, enabling long‐term maintenance of DNA concentration over continuous cycles of DMF‐based artificial cell replication.

## 3 Discussion

Together, these results demonstrate that DMF can be used as a platform to mimic compartmentalized, self‐replication of an artificial cell, with continuous cycles of content amplification. Clearly, this direct control of compartment division and growth is very different to that found in living cells; however, by taking control of this process, we can explore directly how compartmentalization and division affect other processes important for high‐fidelity artificial cell replication, and in this work, we have focused on DNA self‐replication as a simple exemplar.

OpenDrop was previously limited to static assays (e.g., nanocluster self‐assembly,^[^ ^48^ ^]^ in situ spectroscopy^[^ ^49^ ^]^). By optimizing it with thin PVC dielectric films (Figure S1, Supporting Information), a fixed droplet channel (Figure S2, Supporting Information), calibrated resistive heaters (Figure S3, Supporting Information) and droplet division electrode patterns (Figure S5, Supporting Information), we have repurposed it as a testbed for mimicking the cell cycle.

In contrast to previous reports^[^ ^46^ ^]^ we employ mixing throughout RPA amplification. This change improved the cycle‐to‐cycle repeatability with a real‐world coefficient of variation of 4.65% for mixing and division (Figure S8, Supporting Information). We note that DMF RPA amplification in these experiments plateau at ≈50‐min (Figure 3), this is somewhat slower than previous reports, e.g., Kalsi, et al. reported DMF RPA plateauing at approximately halve this time (25 min).^[^ ^46^ ^]^ However, here we also incorporate division steps and longer amplicons (⩽200 bp),^[^ ^62^, ^63^ ^]^ both of which would explain these minor differences. Previous mathematical models indicate that off‐chip RPA reactions can achieve shorter amplification times (Figure S10, Supporting Information), but at the cost of substantial variability.^[^ ^64^ ^]^ Inconsistencies in other off‐chip reactions (e.g. LAMP) have been reported as well.^[^ ^44^ ^]^ Moreover, the DMF approach produced well‐resolved and distinct gel electrophoresis bands (Figure 3d) in contrast to vesicle‐based DNA amplification, where the gel electrophoresis bands of recovered amplified DNA, were poorly resolved.^[^ ^11^ ^]^

While prior DMF studies focused on single cell analysis^[^ ^39^, ^40^ ^]^ or isolated DNA amplification,^[^ ^44^, ^46^ ^]^ our system integrates these processes into a continuous cycle—daughter droplets that inherit parental DNA (Figure 4b) and maintain replication capacity for 20 generations (Figure 4d), a key feature of living systems, and to our knowledge, previously unreported using DMF. In contrast, Ruan et al.^[^ ^45^ ^]^ amplified single‐cell DNA in DMF droplets, and Kalsi et al.^[^ ^46^ ^]^ used DMF for gene detection, with no division‐replication coupling. We have achieved reliable artificial cell division and growth, where the replication activity increased after every cycle, with SD reaching no more than 9 % by cycle 20. Comparing this to the vesicle‐based replication by Abil et al, no prolonged cyclic improvement of self‐replication capabilities was observed during intermittent evolution^[^ ^21^ ^]^ and only 50 % of their samples (3 out of 6) demonstrated five‐fold amplification by the final cycle, with SD exceeding 60 %. Due to the controlled growth and division, our technique conveys a reliable method for DNA‐self replication and growth without loss of replication activity.

Existing artificial cell replication relies on complex biochemical mechanisms (e.g., phase separation^[^ ^10^ ^]^) with poor division control. Upon addition of Mg(OAc)_2_, controlled cyclic replication was established, resulting in a 7.5‐fold enhancement of the signal by cycle 5 (Figure 4b), whilst Abil et al. reported neutral shift over 6 cycles upon the addition of vesicles encapsulating replication additives.^[^ ^21^ ^]^ Unlike the random vesicle fusion, the controlled merging and division of our DMF methodology, ensures accurate provision of components to achieve amplification. Our system uses EWOD actuation to achieve uniform droplet division (CV <5%, Figure S8, Supporting Information) and temperature‐tunable replication cycles (Figure 4d), avoiding reliance on complex biochemical networks. This balances simplicity and precision—an advantage over vesicle‐based systems^[^ ^10^ ^]^ that lack timing or size control during division.

## 4 Conclusion 

Overall, this work establishes DMF as a powerful platform for creating artificial cells with controlled cycles of replication and division ‐ two defining features of living systems. By decoupling the biochemical complexity of natural cells from their essential physical processes, our approach offers a framework to explore how genetic replication can be coordinated with compartment division for the successful propagation of information. Our approach is also a suitable analogue for early self‐replicating systems that may have relied more on physical principles than biochemical sophistication, before evolving the elaborate protein machinery seen in modern cells. Our platform enables control over experimental parameters relevant for investigating the emergence of evolutionary behaviors, including manipulation of selection pressures, inheritance patterns, and mutation rates; capabilities that are challenging to access in conventional artificial cell systems. Although here we have focused on robust, reproducible readout of DNA, we envisage an on‐chip readout, such as fluorescence, would enable cyclic rounds of selected evolution using this approach. By providing a reliable, programmable testbed for studying the requirements of self‐replicating systems, this work opens new avenues for exploring the boundaries between non‐living and living matter ‐ providing an alternative route to circumvent many of the limitations of membrane‐containing and membrane‐less systems in studying the mechanics of replication relevant to artificial cells.

## 5 Experimental Section

### Materials

OpenDrop V4 driver board, drilled ITO cover glasses, and cartridge frames were purchased from GaudiLabs LLC (Switzerland). FluoroPel PFC1601V was purchased from CYTONIX (USA). Octamethyltrisiloxane was obtained from Fluorochem Limited (UK). Methylene Blue hydrate (⩾95%), Tartrazine (⩾85%), Tris(hydroxymethyl)aminomethane (⩾99.8%), and silicone oil AR5 (5 cSt at 25 °C) were purchased from Sigma–Aldrich (UK/USA). Fluorescein (free acid) was obtained from Fluka Chemie GmbH (UK). TwistAmp exo and basic RPA kits were purchased from TwistDx (USA). pUC19 was obtained from New England BioLabs (UK).

### OpenDrop Device Setup and Operation

The OpenDrop device comprises of a 14 × 8 array of gold‐coated electrodes (2.75 mm × 2.75 mm, with 4 mil gaps) and integrated resistive heating elements. The platform was operated in alternating current (AC) mode at 160 V and 350 Hz for droplet manipulation experiments, and at 50 V and 100 Hz for heating operations. Each electrode accommodated a 2 µL droplet volume, with reservoirs holding 16 µL. Modifications to the standard device were made to enable effective DMF‐based artificial cell replication as detailed in the Supporting Information.

### Serial Cyclic Dilution Setup and Operation

Reservoir A and B were injected with 16 µL of methylene blue (1 mM, ultra‐pure water, 1% v/v silicone oil AR5) and 16 µL of tartrazine (1 mM, ultra‐pure water, 1% v/v silicone oil AR5), respectively. The programmed pattern consisted of five steps: (i) Initiation, (ii) Mixing, (iii) Division, (iv) Growth and (v) Readout. The droplet for cycle 0 was a 1 mM tartrazine solution, and the droplets for cycles one to seven were continuously diluted with 1 mM methylene blue solution.

For sequential droplet growth and division experiments, Reservoir A was filled with 16 µL of fluorescein (75 nM in 0.1 M Tris‐HCl buffer, pH 8.0) containing 1% (v/v) silicone oil AR5, and Reservoir B with 16 µL of 0.1 M Tris buffer (pH 8.0) containing 1% (v/v) silicone oil AR5. The droplets were sequentially analyzed using a fluorometer (Cary Eclipse, excitation wavelength 490 nm, emission wavelength 513 nm, excitation slit 5 nm, emission slit 5 nm, PMT voltage 1000 V).

### Real‐Time RPA Reaction on DMF Setup and Operation

RPA mixture was prepared in a 2 mL sterile microcentrifuge tube, consisting of 1.68 µL of 10 µM forward primer, 1.68 µL of 10 µM reverse primer, 0.48 µL of 10 µM probe, 5.6 µL of rehydration buffer, 0.8 µL of 8.06 nM positive control template, 7.76 µL of autoclaved water, and 2 µL of 1% Tween 20 solution. The 20 µL RPA mixture was centrifuged at 7,000 rpm (3,200 × g) for 90 s, after which it was carefully introduced to a lyophilized enzyme pellet. This preparation was centrifuged at 6,000 rpm (2,000 × g) for 3 min and added to reservoir A.

A diluted Mg(OAc)_2_ solution was prepared by mixing 2 µL of 280 mM Mg(OAc)_2_ with 18 µL of rehydration buffer, centrifuged at 7,000 rpm (3,200 × g) for 90 s and loaded into reservoir B. The chip was filled with 400 µL of octamethyltrisiloxane. Droplets were distributed from reservoirs A and B, mixed for 4 min, and heated to 40 °C. Samples were collected at specified time points, placed on ice to quench the reaction, and analyzed by fluorescence or gel electrophoresis.

For gel electrophoresis analysis, a 277 bp segment of pUC19 was amplified using a TwistAmp Basic kit with primers F41 (5'‐GGGTAACGCC  AGGGTTTTCC  CAGTCACGAC  GTTGTAAAAC  G‐3') and R‐43‐mer (5'‐ACAGGTTTCC  CGACTGGAAA  GCGGGCAGTG  AGCGCAACGC‐3'). Products were analyzed on a 1.5% (w/v) agarose gel containing SYBR Gold at 60 V for 90 min.

### Regulation of Sustained DMF DNA Self‐Replication

For Mg^2 +^/EDTA switching experiments, a RPA mixture was loaded into reservoir A (containing primers, probe, rehydration buffer, Tween 20 and lyophilized enzymes), while reservoir B contained either 56 mM Mg(OAc)_2_ or 160 mM EDTA solution. DNA template (0.32 nM) was added to reservoir C. In cycle 0, droplets of DNA solution were mixed with RPA mixture and Mg(OAc)_2_ without amplification, divided into two equal volumes, with one half collected for analysis and the other entering cycle 1. In subsequent cycles, Mg(OAc)_2_ was supplied in cycles 1–5 and 11–15, while EDTA was supplied in cycles 6–10 and 16–20. Each cycle was heated at 40 °C for 15 min.

For temperature‐switching experiments, reaction temperature alternated between 25 °C for odd‐numbered cycles and 45 °C for even‐numbered cycles, with Mg(OAc)_2_ supplied in every cycle. DNA template (3.2 nM) was added to reservoir C. The DMF platform operated at 160 V at 200 Hz for droplet manipulation and 50 V at 100 Hz for heating. All experiments were performed in triplicate, with samples analyzed using a fluorometer (Cary Eclipse, excitation wavelength 488 nm, emission wavelength 520 nm, excitation slit 10 nm, emission slit 10 nm, PMT voltage 900 V).

### Statistical Analysis

### Statistical Analysis—Pre‐Processing of Data

Spectral analysis of the methylene blue‐tartrazine cyclic mixed droplets (Figure 2b) was conducted between 350–740 nm, covering the characteristic absorption range of both dyes while excluding irrelevant baseline noise. The signals of undiluted samples initially exceeded the spectrophotometer's detection limit. This issue was addressed by preparing samples via a 1:1 volumetric dilution prior to measurement. To correlate the readings back to the original concentration, all measured absorbance values of the diluted samples were multiplied by a correction factor of 2.

For fluorescein concentration analysis in cyclic droplet replication (Figure 2e), maximum fluorescence intensities of droplets from Cycles 0–20 were converted to measured concentrations using the pre‐established linear calibration curve (Figure S6b, Supporting Information, *y* = 15.6467*x* + 12.4396, *R* ^2^ = 0.9970). Theoretical concentrations for Cycles 1–20 were calculated based on the known initial fluorescein concentration of Cycle 0 (75 nM) and the expected serial dilution factor of each cycle (determined by droplet volume ratios during mixing and division).

To address saturation at the 1000 a.u. detector limit, samples from Figure 4d (cycles 0–20) were first measured at 900 V. Any signal reaching 1000 a.u. was re‐measured at 850 V. These 850 V values were then converted to their 900 V‐equivalent intensities by multiplying by a calibration factor of 1.639, which was established from prior comparative measurements of identical samples at both voltages. Unsaturated samples at 900 V required no correction.

### Statistical Analysis—Data Presentation and Sample Size

Quantitative data are presented as mean ± standard deviation (SD) throughout the manuscript, unless otherwise noted in the figure legends. The sample size (n) for each experiment represents the number of independent experimental replicates. The specific replicates for each dataset are as follows:

i) The measured fluorescein concentration in the cyclic replication experiments (Figure 2e; Figure S7, Supporting Information). *n* = 3 independent experimental replicates.
ii) The calibration data for the chip heater and standard fluorescein solutions (Figure S6b, Supporting Information). *n* = 3 independent experimental replicates.
iii) The fluorescence intensity measurements from both on‐chip (Figure 3c) and off‐chip RPA reactions (Figure S10, Supporting Information): *n* = 3 independent DMF RPA reactions per time point (0, 10, 20, 30, 40, 50, 60 min).
iv) The fluorescence intensity measurements of the regulation of the DNA self‐replication, periodic addition of Mg(OAc)_2_ and EDTA (Figure 4b; Figure S11, Supporting Information) and temperature‐switching experiments (Figure 4c,d): *n* = 3 independent replicates per temperature group (25, 30, 35, 45, 55 °C) and per cycle (0–20 cycles).
v) Droplet division uniformity (Figure S8, Supporting Information): n = 5 independent maximum fluorescence measurements of the daughter droplet from reproducible mixing and division of fluorescein droplets.

### Statistical Analysis—Software Used for Statistical Analysis

All statistical calculations and data visualization were performed using Python 3.13.2. Raw data were organized and pre‐processed using Microsoft Excel 16.0.4266.1001 (Microsoft Office Professional Plus 2016) before statistical analysis.

## Author Contributions

G.Z. designed and performed all experiments, analyzed the data, and drafted the manuscript. P.D. assisted with data analysis and manuscript preparation. J.S. established the original OpenDrop platform and provided technical support. I.W. conceived the project, secured funding, supervised the research, and finalized the manuscript. All authors reviewed and approved the final version.

## Conflict of Interest

The authors declare no conflict of interest.

## Supporting information

Supporting Information

Supporting Information

## Data Availability

The data that support the findings of this study are openly available in https://doi.org/10.18742/30384391.
