# Supplementary figures and images for: Orchestrating Self‐Replication in Artificial Cells with Digital Microfluidics
Source: Small. 2025 Nov 18;21(50):e09316. doi: 10.1002/smll.202509316 (PMC12710134; doi:10.1002/smll.202509316)

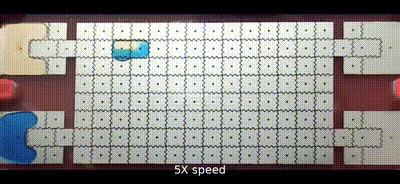

Supplement: Supplementary file 2 — Supporting Information [file SMLL-21-e09316-s001.gif]
